# Evaluation of an ivermectin-based attractive targeted sugar bait (ATSB) against *Aedes aegypti* in Tanzania.

**DOI:** 10.12688/wellcomeopenres.17442.1

**Published:** 2022-01-05

**Authors:** Frank Sandra Chelestino Tenywa, Jeremiah John Musa, Revocatus Musyangi Musiba, Johnson Kyeba Swai, Ahmad Bakar Mpelepele, Fredros Okech Okumu, Marta Ferreira Maia

**Affiliations:** 1 Environmental Health and Ecological Sciences Thematic Group, Ifakara Health Institute, Bagamoyo, Pwani, 0000, Tanzania; 2 Vector Biology, Swiss Tropical and Public Health Institute, Basel, Basel, CH-4002, Switzerland; 3 Science, University of Basel, Basel, Basel, CH-4002, Switzerland; 4 Faculty of Health Science, School of Public Health, University of the Witwatersrand, Johannesburg, Johannesburg, 0000, South Africa; 5 Wellcome Trust Research Program, Kenya Medical Research Institute(Kemri ), Kilifi, Mombasa, 0000, Kenya; 6 Medicine, Centre for Global Health and Tropical Medicine, University of Oxford, Oxford, OX3 7FZ, UK

**Keywords:** Attractive-targeted sugar bait, ivermectin, Aedes aegypti, vector control, Dengue fever, Tanzania

## Abstract

Background

The control of vector borne arboviral diseases such as Dengue is mainly achieved by reducing human-vector contact and controlling the vectors through source reduction and environmental management. These measures are constrained by labour intensity, insecticide resistance and pro-active community participation. The current study intended to develop and test an ivermectin-based attractive-targeted sugar bait (ATSB) against *Aedes aegypti*.

Methods

The 48hour lethal concentration (LC90) of ivermectin against *Ae. aegypti* was determined through serial dilution experiment where five 30cm x 30cm x 30cm cages were set; into each, a 10% sugar solution treated with ivermectin were introduced. 40 *Ae. aegypti* were released into each cage and observed for mortality after 4, 8, 24 and 48 hours. The ivermectin-based ATSB was evaluated in a semi field system where ATSB and attractive sugar bait (ASB) were deployed into each compartment of the semi field and 100 female *Ae. aegypti* were released every day and recaptured the next day through human land catch and Bio-gent sentinel trap. The developed and semi-field tested ATSB was further tested in the field by deploying them in garages.

Results

The ivermectin 48hr LC90 of male and female *Ae. aegypti* was found to be 0.03% w/v. In the semi field system, the ATSB significantly reduced a free-flying population of *Ae. aegypti* within 24 hours (incidence rate ratio (IRR) = 0.62; [95% confidence interval (95%CI); 0.54-0.70] and p-value < 0.001). However, in the field, the ATSBs required the addition of yeast as a carbon dioxide source to efficiently attract *Ae. aegypti* mosquitoes to feed.

Conclusion

Ivermectin is an active ingredient that can be used in an ATSB for *Ae. aegypti* depopulation. However, further research is needed to improve the developed and tested ATSB to compete with natural sources of sugar in a natural environment.

## Introduction

Dengue fever is a global public health concern estimated to threaten 100 to 200 million people per year worldwide, with 2.5 billion people worldwide approximated to be at risk ( World Health Organization *et al.*, 2009). The disease is endemic in 100 countries, with Asian and South American countries being the most affected ( Kyle & Harris, 2008). The disease is reported to have increased by 30-fold in the past half century ( World Health Organization, 2012) and is projected to increase further in the coming years ( World Health Organization [WHO], 2020a). Reports from Bhatt *et al.*, 2013 have shown that the disease is spreading globally and can no longer be viewed as a regional problem, with cases occurring in countries without past history of disease outbreaks ( Bhatt *et al.*, 2013), including Tanzania ( Ward *et al.*, 2017).

The primary and secondary vectors of the dengue fever are *Aedes aegypti* (L) and *Aedes albopictus* (Skuse), respectively ( Barrett & Higgs, 2007). They are widely distributed all over the world as a result of the rapid expansion of poorly planned cities, lack of piped water systems, poor sewage systems, high urban population growth ( Gubler, 2011; Guzman & Harris, 2015) and the ability of the vectors to oviposit in diverse environments ( Gómez-Dantés & Willoquet, 2009; Hawley, 1988). Additionally, an increase in inter-continental travel involving trade and tourism ( Messina *et al.*, 2019) has largely contributed to spread of the vectors as well as the disease.

For decades, there have been no effective recommended antiviral drugs for dengue fever treatment. However, in 2015, WHO recommended the use of the CYD-TDV vaccine (tetravalent dengue vaccine) against dengue fever for travellers going to countries with a seroprevalence of 70% and above ( Wilder-Smith *et al.*, 2019). In 2019, the United States Food and Drug administration (FDA) approved the vaccine for individuals residing in endemic countries ( FDA Commissioner, 2019). Nonetheless, the vaccine protects only individuals who are seropositive (individuals who had a previous infection) and not seronegative ones ( Sridhar *et al.*, 2018). Thus, disease prevention continues to heavily rely on vector control, mainly through source reduction (larviciding and environmental management), preventing human-vector conduct (repellent and insecticide treated materials) and adult depopulation (insecticide space spraying, indoor space spraying) ( Gómez-Dantés & Willoquet, 2009; Ranson *et al.*, 2010; Vontas *et al.*, 2012; World Health Organization, 2009). However, current control measures require untiring commitment from health authorities and community members, and is challenged by economic constraints in low- and middle-income countries (LMIC). In addition, insecticide-based tools are threatened by the emergence of insecticide resistance ( Lima *et al.*, 2011; Ponlawat *et al.*, 2005; Rodríguez *et al.*, 2007; Vontas *et al.*, 2012). The development of a low-cost, peri-domestic intervention such as an attractive-targeted sugar bait (ATSB) against *Aedes* mosquitoes could have the potential to expand the intervention toolbox and improve disease control.

In recent years, ATSBs have surged as a novel vector control paradigm ( Beier *et al.*, 2012; Müller *et al.*, 2008; Müller *et al.*, 2010a; Naranjo *et al.*, 2013; Qualls *et al.*, 2015b). The intervention exploits mosquito sugar feeding behaviour, which is common to both male and female mosquitoes. Usually male mosquitoes exclusively feed on sugar for their whole life while females feed on sugar for survival, flight and fecundity enhancement ( Foster, 1995) but require blood for egg-laying. Therefore, an intervention that targets this behaviour will concurrently target both male and female mosquitoes. ATSBs have been researched for controlling malaria vectors and have successfully reduced the vector populations ( Müller *et al.*, 2008; Müller *et al.*, 2010b). Additionally, they have also been found to impact the density and fitness of *Aedes* mosquitoes ( Ali *et al.*, 2006; Xue *et al.*, 2006) as well as other sugar-questing vectors such as sand flies ( Qualls *et al.*, 2015a; Saghafipour *et al.*, 2017). Furthermore, ATSBs appear to reduce human landing rates ( Junnila *et al.*, 2015; Xue *et al.*, 2006; Xue & Barnard, 2003), a parameter that highly influences disease transmission

Ivermectin was selected as the ATSB toxicant because of its holistic ability to reduce densities, fitness and virus replication in the mosquitoes. Ivermectin has been reported to reduce mosquito survival ( Chaccour *et al.*, 2010), inhibits oviposition ( Focks *et al.*, 1991) as well as reducing viral load of flaviviruses inside the mosquitoes including dengue viruses ( Mastrangelo *et al.*, 2012; Wagstaff *et al.*, 2012; Xu *et al.*, 2018). In addition, ivermectin has a different mode of action: it targets the glutamate-gate chloride channel compared to other insecticides commonly used for vector control ( Hemingway & Ranson, 2005), a property that may circumvent the existing resistance mechanisms in the mosquitoes ( Foy *et al.*, 2011). Ivermectin also has a good safety record for use in humans and other mammals making it suitable for a bait solution in case of accidental ingestion. For more than three decades, it has been distributed in mass drug administration (MDA) campaigns against filariasis and onchocerciasis with over one billion doses donated by the Mectizan project ( Crump & Omura, 2011). The safety of the endectocide is likely related to its target receptors in arthropods where upon being ingested, the ivermectin directly targets glutamate-gated chloride channels (GluCl), a receptor channel which exists in invertebrate nerve and muscle cells leading to paralysis and death of the insect ( Fox, 2006; Sheriff *et al.*, 2005) but is non-existent in vertebrates. This study aimed at evaluating the efficacy of an ivermectin-based ATSB as a potential approach for controlling *Ae. aegypti* mosquitoes in urban Tanzania.

## Methods

### Mosquitoes

All experiments were conducted at Ifakara Health Institute Bagamoyo and Ifakara branches using disease free, insectary-reared *Aedes aegypti.* The mosquitoes were reared at 27±5 ^0^C and 40%-99% humidity at Ifakara Health Institute insectary in Bagamoyo and Ifakara, Tanzania. Larvae were fed daily with Tetramin ^®^ fish food while adults were maintained with 10% w/v sugar solution ad libitum with natural light regimen. For egg laying, they were fed cow blood through a membrane.

### Laboratory experiment

***Determination of ivermectin 48hr LC90 for Ae. aegypti.*** The dose of ivermectin sufficient to kill 90% of *Ae. aegypti* was determined in a laboratory using insectary-reared mosquitoes. Serial dilutions of ivermectin in 10% w/v sugar solution were obtained starting with 0.01% of ivermectin as a starting point following a report by Tenywa *et al.* ( Tenywa *et al.*, 2017) that found 0.01% of ivermectin in 10% w/v sugar solution was enough to kill more than 90% of malaria vector ( *Anopheles arabiensis*) within 48 hours. To make a 0.01% ivermectin solution, 1ml of 1% injectable ivermectin (Ivomec®) was diluted in 100ml of 10% w/v sugar solution. The procedure was repeated to obtain other ivermectin concentrations: 0.001%, 0.0025%, 0.005%, 0.015%, 0.02%, 0.025%, 0.03%, 0.04%, 0.05% and 0.06%. The solutions were dyed with a food colouring dye (Carmoisin) at 0.5% v/v concentration for easy visualization of sugar fed mosquitoes.

Experiments were done using two rounds of five cages (30cm × 30cm × 30cm) placed inside the insectary. Each ivermectin concentration was poured into a 30mL plastic container to 2/3 of its capacity. These containers are regularly used for delivering glucose to mosquitoes reared in the insectary. A Whatman paper (filter paper) was rolled up like a tube and then dipped into each container containing the sugar solution with ivermectin concentration. The sugar solutions were randomly placed into each of the 30cm × 30cm × 30cm cages without blinding the researchers. One cage with 10% w/v sugar solution without ivermectin served as a control for the experiment. The sugar solution progressed up the filter paper where the mosquitoes had access to it. 40, 3–6 days old, blood naïve and starved for 6–8 hours mosquitoes were introduced into each cage and allowed to feed on the soaked filter paper dipped into the container containing ivermectin in 10% sugar solution. For an entire experiment, a total of 2200 mosquitoes were used as per ( Tenywa *et al.*, 2017). Mosquito mortality was recorded after 4-, 8-, 24- and 48-hours post-introduction of the treatments. For each ivermectin concentration, five experiments (replicates) were performed while changing the position of the cages after each experimental replicate to avoid bias due to cage positioning.

Similar experimental set up and procedures as described above were performed for male *Ae. aegypti* where eight ivermectin doses were tested: 0.005%, 0.01%, 0.015%, 0.02%, 0.025%, 0.03%, 0.04% and 0.05%. In this round of the experiment, 1800 mosquitoes were used.

### Semi field experiment

***Efficacy of ATSB on population density and survival of Ae. aegypti in a controlled environment.*** The efficacy of ATSB was determined in a semi field system at Ifakara Health Institute’s mosquito city in Ifakara. The semi field system (biodome) is 19m × 29m long; sitting on a concrete slab and surrounded with a water moat which prevents mosquito predators from entering the biodome. Its floor is of mud and the walls are made of net which allows ambient conditions inside the biodome to be similar to the outside environment. The biodome has two compartments separated by a 4m × 29m corridor and is roofed with polyvinyl sheets ( Ferguson *et al.*, 2008). A hut mimicking rural houses in terms of doors, windows, eaves, structure and size is installed in each of the two biodome compartments. The huts’ are made of red brick walls and grass roofs. To reduce the temperature inside the biodome that could lead to mosquito desiccation the biodome was modified by adding a thatch layer beneath the polyvinyl roof. The entry doors were sealed with double nets and fixed with a zip in order to prevent mosquitoes from escaping the system when a person entered or exited the biodome.

The two compartments of the biodome were labelled as A and B. Five control-ATSB (ATSB without ivermectin) and ATSBs were made following Tenywa *et al.*, ( Tenywa *et al.*, 2017) and deployed into each of the compartments. 100 *Ae. aegypti* mosquitoes aged 2 – 5 days, blood naïve and starved for 6–8 hours were released into each compartment at 6:00 am every day consecutively for 30 days, simulating every day emerging mosquitoes in the wild population. In compartment A, the five control-ATSBs were deployed in all 30 days of the experimentation while in compartment B, the 30 days of experimentation were done in three phases each of 10 days. During phase I (day 0 to 10 of experimentation) and phase III (day 21 to 30 experimentation) control-ATSB were placed in the compartment, while during phase II (day 11 to 20 of experimentation) the control-ATSB were replaced with ATSBs containing 0.03% ivermectin. To maintain quality of the control-ATSB and ATSB, both were replaced with a new one after every five days.

24 hours after every mosquito release, one Biogent (BG) sentinel trap was deployed into each compartment for three hours from 6:30am to 9:30am to collect mosquitoes for monitoring the *Ae. aegypti* population density. Mosquitoes collected from each compartment were killed with ethanol by spraying them, counted and recorded.

**Figure 1.**
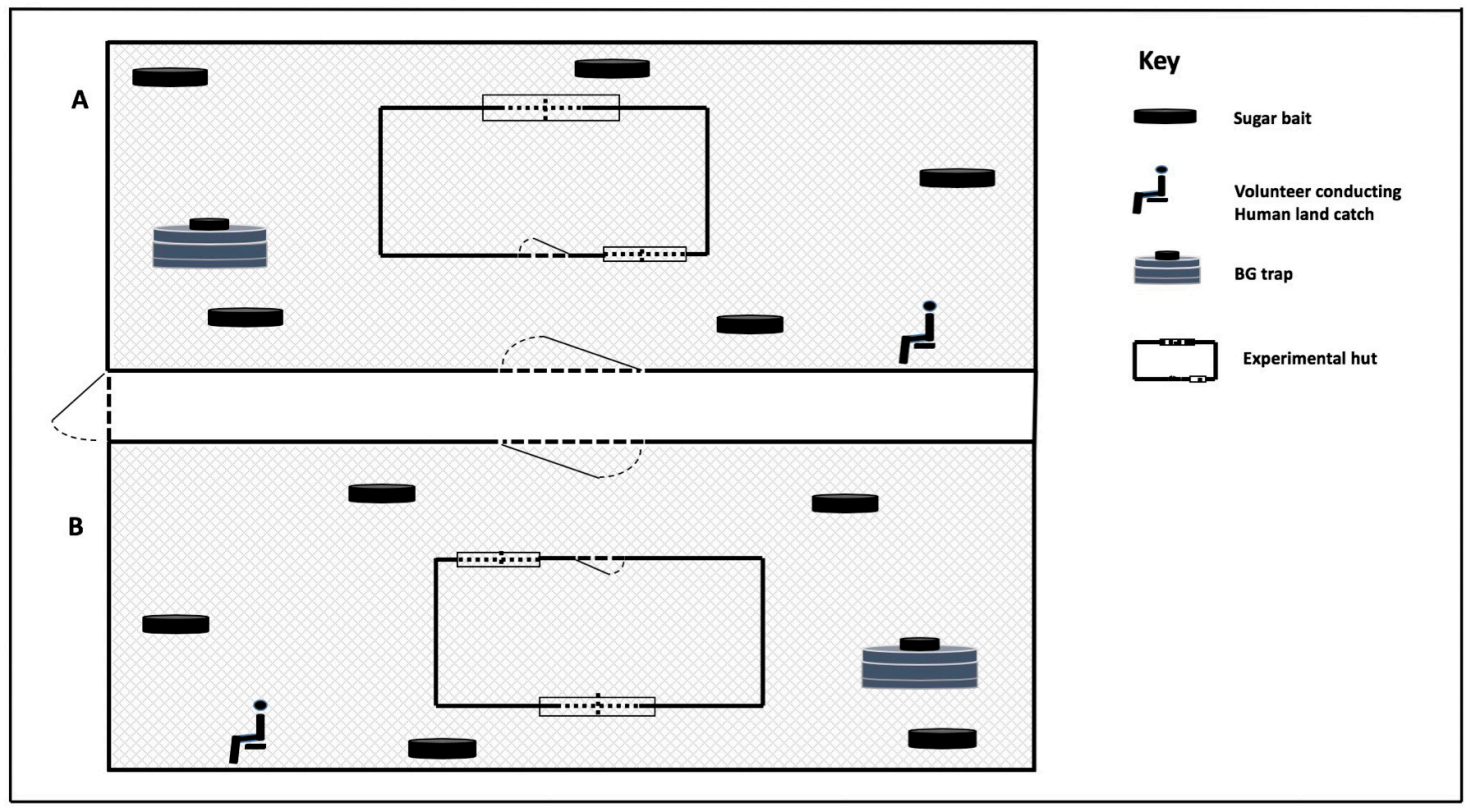
Illustration of attractive targeted sugar bait (test-ATSB or control-ATSB) stations deployed in a semi field. The control-ATSB and test-ATSB were deployed into either compartments A or B for 30 days with replacement of the control-ATSB by test-ATSB at day 10-20 in compartment B to determine its efficacy against *Ae. aegypti* population density in a controlled environment. One Bio-gent sentinel trap was deployed into each semi field compartment every day at 6:30am – 9:30am for 30 days. A volunteer conducted human land catch in each compartment from day 11 to 20 of the experimentation for 20 minutes from 6:00am to 6:20 am.

During phase II, human landing catches (HLC) were done in compartment A and B for 20 minutes from 6:00am to 6:20am before deployment of the BG sentinel trap at 6:30am to assess the impact of ATSB on *Ae. aegypti* survival. One volunteer wearing shorts, closed shoes, a long sleeve shirt and hat, sat inside each of the semi field compartment and conducted a human land catch to collect mosquitoes landing on his shins ( Figure 1). To reduce bias due to differences in volunteer attraction to the mosquitoes, the volunteers were swapped every day between the two compartments. All collected mosquitoes from each compartment were kept in paper cups labelled with the collection date and compartment/treatment. The collected mosquitoes were transferred to the insectary and given 10% w/v sugar solution that is used for regular colony maintenance. Sugar was delivered by soaking a small ball of cotton wool into 10% w/v sugar solution and put on top of the netting covering the paper cup lid. Daily mortality was recorded for 30 days. After the 30 days, the mosquitoes that remained alive were sprayed with ethanol and discarded.

### Field experiment

**Figure 2.**
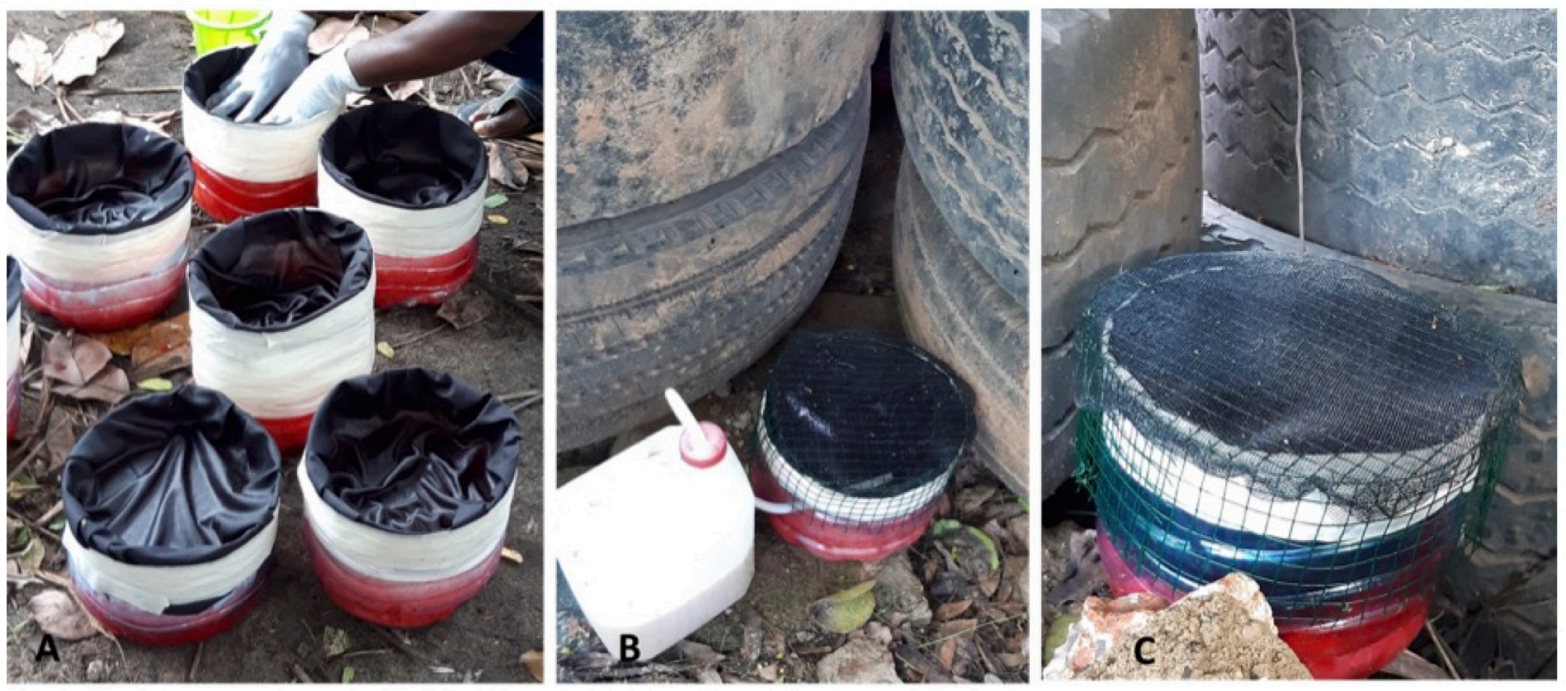
Attractive targeted sugar bait stations (test-ATSB stations). **A**) test-ATSB station without a wire mesh covering the lid; **B**) test-ATSB station with a glue painted wire mesh covering the lid and carbon dioxide source; **C**) test-ATSB station with a glue painted wire mesh covering the lid.

***Preparation of attractive targeted sugar bait (ATSB) with and without a carbon dioxide source.*** ATSB were prepared following procedures described previously ( Tenywa *et al.*, 2017) ( Figure 2A). Three test bait stations were prepared as follows: 1) control bait consisted of plain water dyed with 0.5% v/v red food colouring dye; 2) test-ATSB consisted of 10% w/v of sugar solution and ivermectin (final concentration 0.03% w/v); and 3) test-ASTB+CO _2_ consisted of the same bait as test-ASTB but with addition of a carbon dioxide (CO _2_) source. The carbon dioxide was obtained from yeast. For that purpose, a total of 250grams of sugar (sucrose) and 17.5grams of yeast were dissolved into 2.5 litres of water in a 5 litre bottle. The bottle’s lid was drilled to make a small hole where a small pipe was inserted into and connected to the test-ATSB+CO _2_ bait ( Figure 2B). All the baits were covered with a wire mesh that was painted with rat glue (Tack-Tick Stronghold Glue ^®^) to make a sticky trap ( Figure 2C) trapping any mosquito attracted to land and feed on the bait.

***Application of ATSB in a field setting.*** Field experiments were conducted between November and December 2019 in Tegeta, Kinondoni district in Dar es Salaam; the largest city and economic centre of Tanzania. The city is located at 6.48’S and 39.17’E along the Indian Ocean coast with 1100mm annual rainfall. It is a hub for international travel into and out of Tanzania and experiences frequent dengue outbreaks ( Vairo *et al.*, 2016). A survey was conducted to identify *Ae. aegypti* breeding sites using BG sentinel traps deployed twice a day: 7:00am – 11:00am and 4:00pm – 7:00pm. Two vehicle garages with old used tires were identified as appropriate study sites and labelled as garage A and garage B. The garages were approximately 100 metres away from each other.

Three test-ATSB and test-ATSB+CO _2_ stations prepared as described above were deployed at either garage A or garage B. The stations of the same treatment were placed at 10 metres apart. To each treatment arm (test-ATSB and test-ATSB+CO _2_), three control baits: a bait that consisted of plain water dyed with 0.5% v/v red food colouring dye, were placed; each at three metres away from each treatment station. The treatment and control stations were left at the study sites for 24 hours. After 24 hours, the baits were checked for the presence of the mosquitoes that stuck on the bait station traps, removed and morphologically identified. In order to account for any variations due to difference in mosquito densities between the study sites, the test-ATSBs and test-ATSBs+CO2 were swapped after every 24 hours between the sites. The experiments ran for a total of 12 days. And after every three days, the baits (test-ATSBs and test-ATSBs+CO2) and control were replaced with a new one so as to maintain the quality of the baits.

### Data analysis

All data obtained were analysed using STATA package (Stata corp, College Station, TX).

### Ivermectin LD90 against *Ae. aegypti*

A mean cumulative proportion mortality of mosquitoes for each ivermectin concentration was determined and compared to control (10% sugar solution) at 4, 8, 24 and 48 hours.

### ATSB efficacy against *Ae. Aegypti* in the semi-field and field

Poisson regression model was employed to compare the number of mosquitoes caught in the semi-field compartments that had ATSBs vs control-ATSBs. Mosquito ages, compartments and day were considered as covariates. For the field test, a negative binomial regression model was performed to compare the number of mosquitoes that quested sugar from test-ASTB+CO _2_ and test-ASTB, and control bait consisted of plain water. The incidence rate ratio (IRR) and 95% confidence interval were obtained from the model.

## Results

### Ivermectin LD90 against *Ae. aegypti*

**Figure 3.**
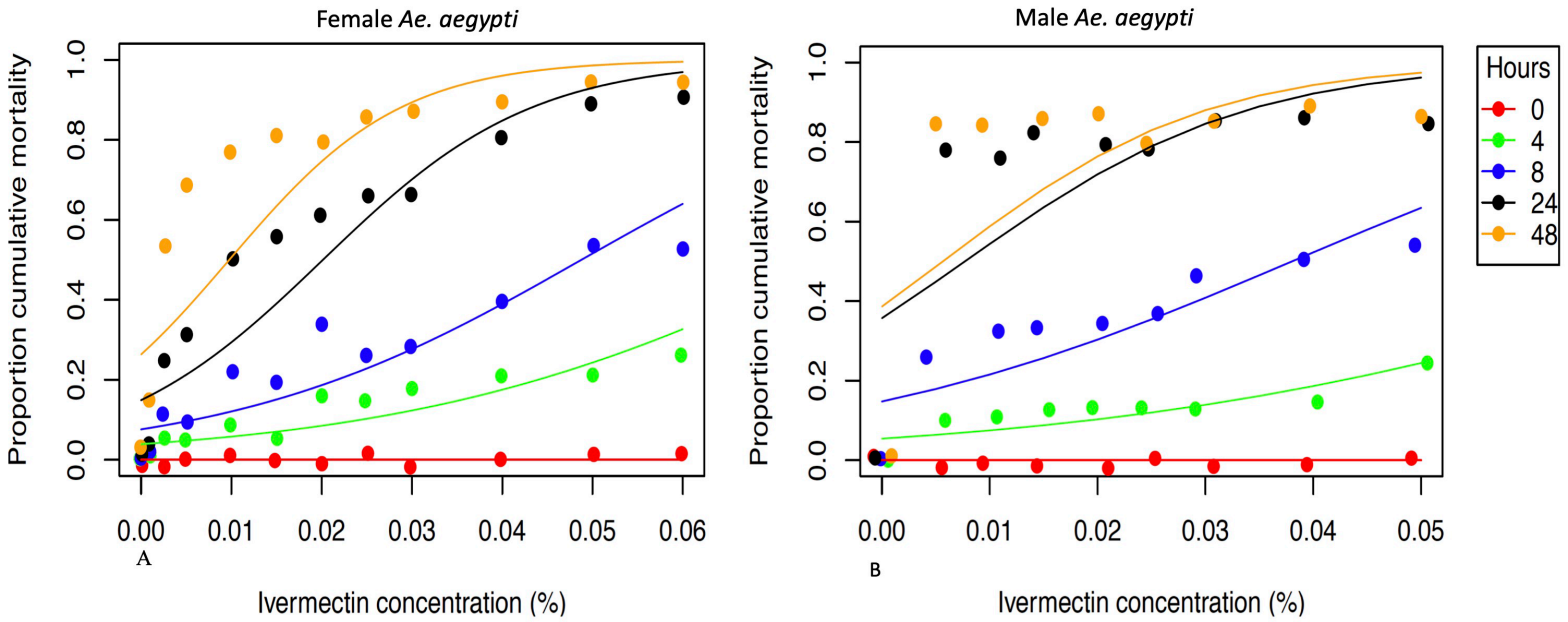
Mean cumulative proportional mortality of female ( **A**) and male ( **B**) *Ae. aegypti* post feeding on 10% sugar solution that contained different ivermectin concentrations.

The results show that ivermectin is toxic to both male and female *Ae. aegypti* ( Figure 3). In insectary conditions, approximately half of female and male *Ae. aegypti* mosquitoes provided with 0.02% (w/v) of ivermectin in 10% sugar solution died within 24 hours. Sugar solution containing ivermectin 0.03% (w/v) caused approximately 80% of mosquito mortality for both mosquito sexes within 24 hours and > 90% within 48 hours ( Figure 3).

### ATSB efficacy against *Ae. aegypti* in a semi field environment

**Figure 4.**
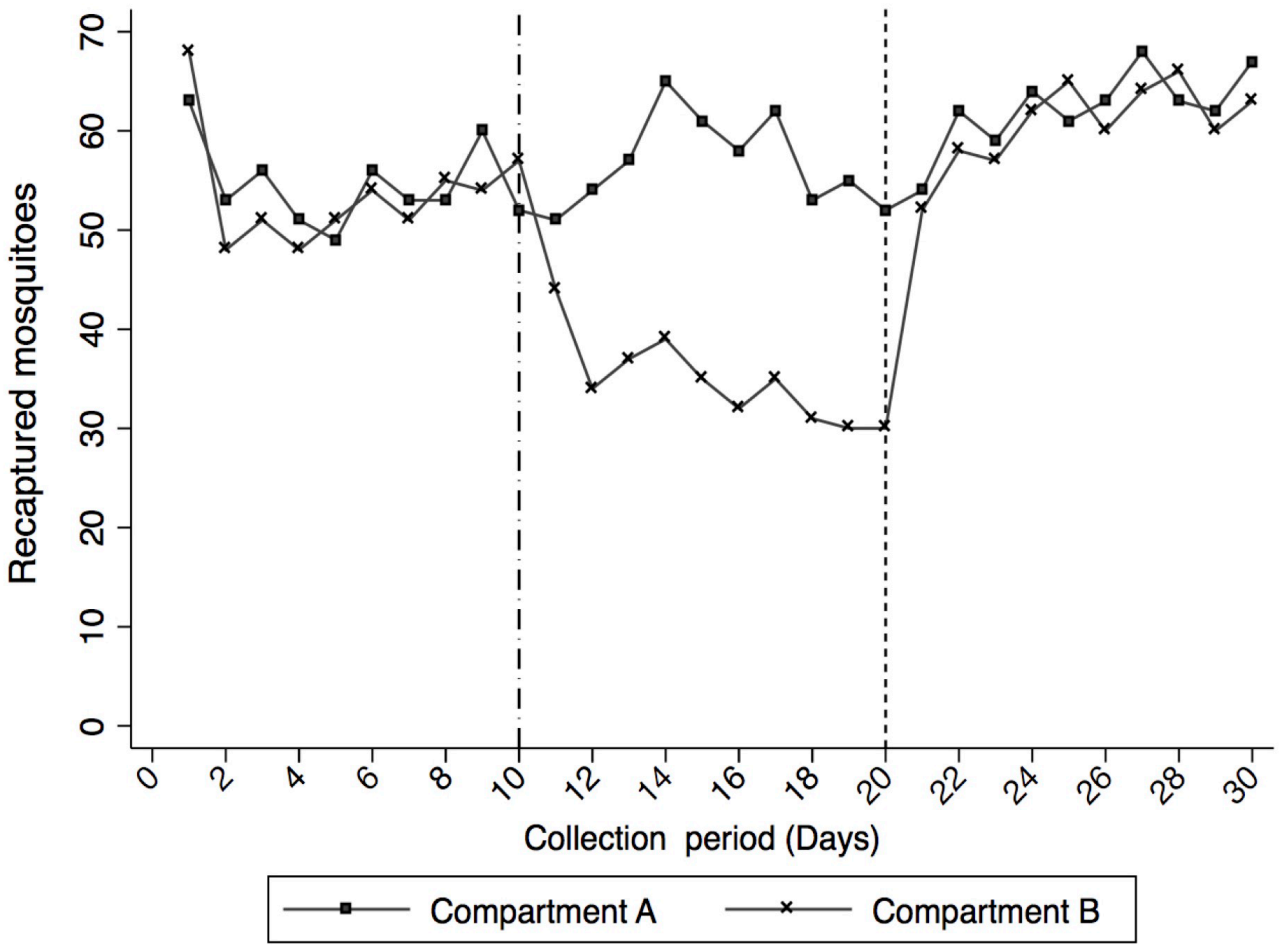
Mosquitoes recaptured in a semi field (controlled environment). **Compartment A** received attractive sugar bait without ivermectin (control-ASB) for 30 days of the experiments and **Compartment B** received ASB in phases I and III (day 0–10 and day 21–30) and in phase II (day 11–20) it received attractive targeted sugar bait with ivermectin (test-ATSB): A *dash dot line* shows when test-ATSB was introduced into the compartment B to replace control-ASB and a *round dot line* shows when the introduced test-ATSB was replaced with control-ASB.

**Figure 5.**
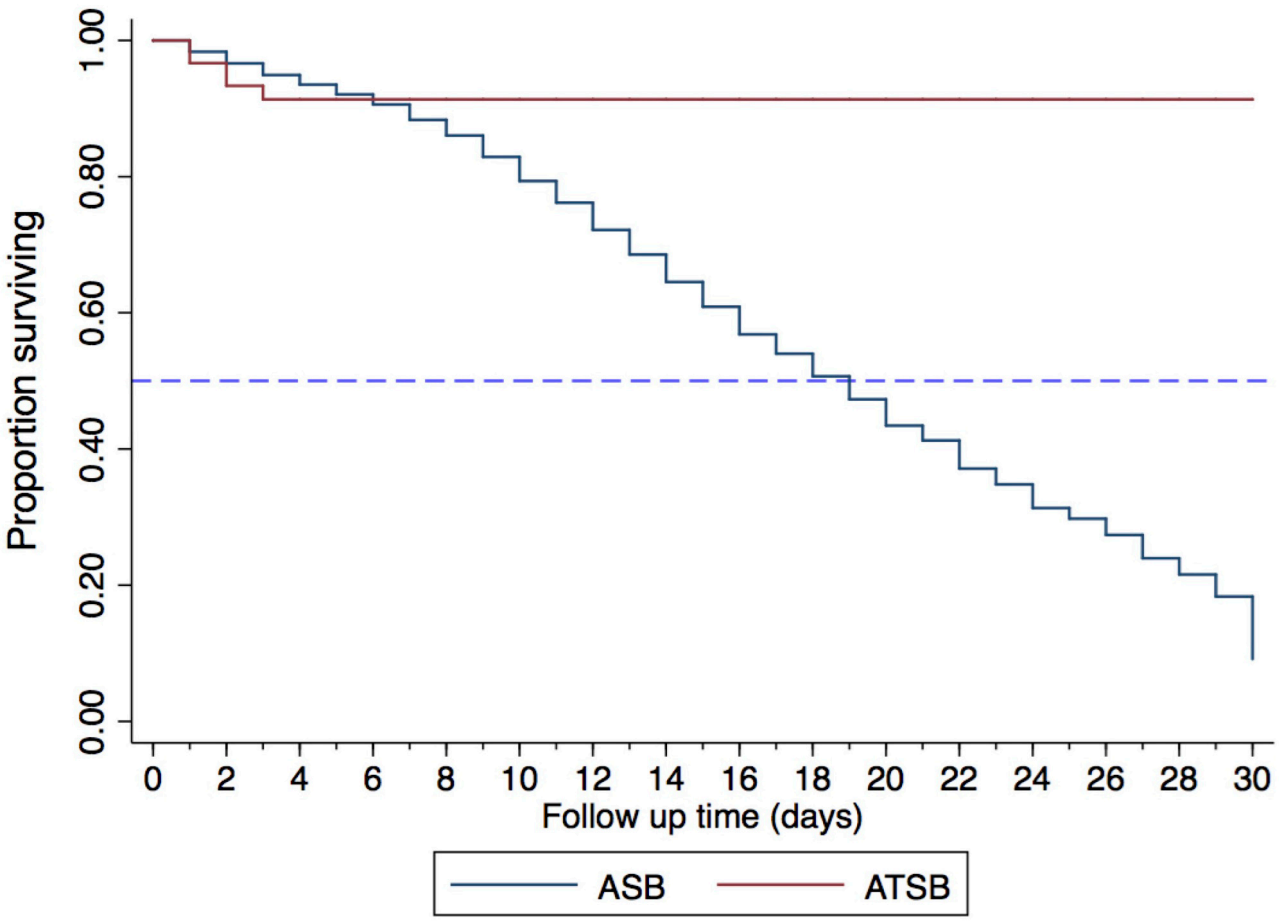
Effect of attractive targeted sugar bait on survival of female *Ae. aegypti* exposed to the baits in a semi-field system for 24 hours.

Mosquito recapture rate using BG-Sentinel traps in compartments A and B of the biodome were comparable before the introduction of ATSBs (incidence rate ratio (IRR) = 0.98; [95%confidence interval (CI): 0.87 – 1.12] and p- value ≤ 0.78) (compartment A, IRR=1). After introducing the ATSBs, mosquito recapture rate inside the compartment was reduced by 38% (IRR = 0.62; [95%CI: 0.54-0.70] and p-value < 0.001) ( Table 1). Approximately 35% (95%CI: 32.0-38.0) and 56.8% (95%CI: 53.5-60.1) mosquitoes were recaptured in compartment with ATSB and control-ATSB respectively ( Figure 4). ATSB reduced *Ae. aegypti* survival time compared to control-ATSB ( Figure 5). Within three days post recapture, 100% (n=253/253) and 7.5% (n= 23/306) of mosquitoes collected from a compartment with ATSB and a compartment with control-ASB respectively died ( Figure 5).

**Table 1.** Cumulative number of recaptured mosquitoes (CR mosquitoes) and incidence rate ratio (IRR) of female *Aedes aegypti* recaptured in a semi field.

Mosquitoes recaptured in semi field compartment with	N	n	CR Mosquitoes	IRR	95%CI	P-value
**ASB**	10	1000	569	1	-	-
**ATSB**	10	1000	350	0.62	0.54-0.70	<0.001

**N** number of replicates, **n** total number of released mosquitoes, **CR-Mosquitoes** cumulative recaptured mosquitoes, **IRR** incidence rate ratio and **95%CI** confidence interval

### ATSB efficacy against wild *Ae. aegypti*

Attractive targeted sugar baits (ATSBs) without carbon dioxide source optimized in a semi field system did not differ in attracting wild *Ae. aegypti* compared to a bait with plain water (IRR = 0.7; [95%CI: 0.36-1.17] and P-value ≤ 0.15) ( Table 2). However, when a carbon dioxide source was added into the ATSB, the incidence rate ratio of captured mosquitoes increased by approximately 4.4 folds (IRR = 6.8; [95%CI:4.11 – 11.30] and P – value < 0.001) in comparison to bait with sugar and ivermect in (IRR = 1) ( Table 2).

**Table 2.** Incidence rate ratio (IRR) of wild female *Ae. aegypti* attracted by ATSB.

ATSB sticky trap	Attracted mosquitoes	IRR	95%IRRCI	P-value
**Bait with sugar and ivermectin**	46	1	-	-
**Bait with plain water (control)**	40	0.7	0.36-1.17	0.15
**Bait with sugar, ivermect in** **and CO _2_ source**	172	4.4	2.52-7.83	<0.001

**IRR** captured mosquito incidence rate ratio, **95% IRRCI** confidence interval.

## Discussion

This study demonstrated that both female and male *Ae. aegypti* are equally sensitive to ivermectin when ingested in a sugar meal. It showed that mosquito mortality is a function of ivermectin concentration and time (ivermectin concentration α mosquito mortality) i.e. the higher the ivermectin concentration the faster the mosquito is killed. Even though *Ae. aegypti* were sensitive to ivermectin, the mosquito species is not as sensitive as other mosquito species such as malaria vectors ( Chaccour *et al.*, 2010; Sylla *et al.*, 2010) which are reported to be sensitive to ivermectin concentrations found in person’s blood after receiving a therapeutic dose. In this study 0.03% of ivermectin killed more than 90% of both *Ae. aegypti* sexes within 48 hours; this dose is three times higher than that which killed the same proportion of female *An. arabiensis* in the same time period ( Tenywa *et al.*, 2017), indicating that this mosquito species is less sensitive to ivermectin.

Moreover, it is known that even low ivermectin concentrations cause sub-lethal effects to mosquitoes including *Ae. aegypti* ( Focks *et al.*, 1991; Tesh & Guzman, 1990) and *Ae. albopictus* ( Tesh & Guzman, 1990) by reducing mosquito longevity, egg hatching rate and shortening the life of mosquito progenies. There may also be effects on the pathogen, as low therapeutic plasma concentration of ivermectin may have anti-dengue viral activity by reducing the ability of the dengue virus to infect mosquitoes ( Xu *et al.*, 2018). Therefore, it may be possible to impact dengue vector populations using lower doses of ivermectin, that may still reach epidemiological significance by reducing vector survival, mosquito fitness and blocking viral transmission to the vectors. In this regard, reducing the ivermectin dose would also likely reduce the risk to non-target species when ivermectin is used in an ATSB whilst keeping its epidemiological impact. Further research is needed to investigate how sublethal doses of ivermectin delivered in sugar solution impact *Ae. aegypti* vectors, and how these effects would impact wild populations densities and transmission dynamics.

This study demonstrated that ATSB deployed inside a semi-field system remarkably reduced *Ae. aegypti* populations within twenty-four hours and had a suppressive effect on the free-flying population. The observed impact of the ATSB stations concurs with Müller *et al.*, 2008 and Xue *et al.*, 2008, which reported a similar impact of ATSB on *Ae. aegypti, Ae. caspius* population as well as *An. segentii* populations. Mosquito survival time for the mosquitoes collected from a compartment which had ATSBs was observed to be less than three days, contrasting with those from the control compartment where approximately half of them survived longer than nineteen days out of thirty days of holding period. The difference in survival time of the mosquitoes from the two groups suggests that the majority of the mosquitoes were attracted by the ATSBs and fed on it.

In the field, the ivermectin-based sugar bait stations (test-ATSB) were inefficient at attracting wild *Ae. aegypti*. The shortcoming of the bait stations is probably attributed by the presence of other natural mosquito meals sources such as plants which were more attractive. The assumption made here corresponds to Xue *et al.*, 2008 which reported similar bait stations’ outcome. Usually sugar baits work on basis of “attract and kill” principal, therefore, the existence of natural sugar sources ( Beier *et al.*, 2012; Müller *et al.*, 2010b) has a critical influence on the bait’s performance. In order for sugar baits to effectively reduce mosquito population in the field, they must release enough attractant volatiles to efficiently attract vectors and there with overcome competition from the natural sugar sources. Also, application strategy (bait stations or vegetation spraying) in the field impacts sugar bait efficiency. Vegetation spraying approach involves spraying the whole vegetations with toxic sugar solution, whereas the bait station approach presents the toxic sugar bait at one single point. In this context, bait station approach may hypothetically be regarded as less effective in attracting mosquitoes due to the fact that mosquito may take a longer time to allocate the solution. However, in spite of the hypothesised limitation, we think that bait stations minimise the possibility of targeting non-targeted organisms such as butterflies, bees, ants and others, making the strategy environmentally safer.

Furthermore, mosquitoes species use cues such as CO _2_ and animal skin odorants to respond towards a host ( Clark & Ray, 2016; Dekker & Cardé, 2011). Carbon dioxide is widely used as lure for mosquitoes trapping ( Cilek *et al.*, 2011; Irish *et al.*, 2008; Meeraus *et al.*, 2008) as it mimicks human odour and receives a potential gain in vector surveillance and control platforms. This study demonstrated that adding carbon dioxide source into the developed ivermectin-based sugar baits increases the attractiveness of the bait against wild *Ae. aegypti.* This finding highlights that carbon dioxide can be a potential component of an ATSB bait to increase attractiveness to mosquitoes in the field.

### Limitation and safety consideration

The ivermectin concentration used in the test-ATSBs in this study is relatively high; although it is unlikely that the bait concoction would be consumed by children, we recommend that further field research done on the baits should investigate these by either placing a protective grill over them or hanging them out of reach.

## Conclusions

Ivermectin-based ATSBs successfully reduce laboratory *Ae. aegypti* populations by approximately 95% within three days in a controlled environment (semi-field) but failed to so against wild *Ae. aegypti* in the field unless a carbon dioxide source was added. We recommend further research to improve bait station design, and attractant strategies such as plant-based volatiles.

### Ethical approval

This study received an ethical approval from Ifakara Health Institute Review Board (IHI-IRB) No. IHI/IRB/No. 22-2017 and National Institute for Medical Research Review Board (NIMR-RB) No. NIMR/HQ/R.8a/Vol. IX/2813. This study was also granted permission to publish from Tanzania National Institute of Medical Research ref No: NIMR/HQ/P.12 VOL XXXIII/85. Furthermore, efforts were made to ameliorate any suffering of the mosquitoes used in the research, especially during euthanasing.

## Data availability

### Underlying data

Open Science Framework: Evaluation of an Ivermectin-based Attractive Targeted Sugar Bait (ATSB) against Aedes aegypti in Tanzania. https://doi.org/10.17605/OSF.IO/3JESA

This project contains the following files:

- ATSB attractiveness against wild Aedes aegypti.xlsx
- ATSB efficacy in a semi field system.xlsx
- Ivermectin dose response_female.xlsx
- Ivermectin dose response_male.xlsx
- Mosquito survival after exposure to ATSB.xlsx

Data are available under the terms of the Creative Commons Zero "No rights reserved" data waiver (CC0 1.0 Public domain dedication).
