# Supplementary material for: Evaluation of an ivermectin-based attractive targeted sugar bait (ATSB) against Aedes aegypti in Tanzania
Source: Wellcome Open Res. 2022 Jan 5;7:4. [Version 1] doi: 10.12688/wellcomeopenres.17442.1 (PMC10318376; doi:10.12688/wellcomeopenres.17442.1)
Supplement: Peer review history

# Peer review: Reviewer response for version 1

**DOI:** 10.21956/wellcomeopenres.19287.r59373

**Published:** 2023-07-05

**Authors:** Rosemary Susan Lees, Daniel McDermott

**Affiliations:** 1 Vector Biology Department, Liverpool School of Tropical Medicine, Liverpool, UK; 2 Clinical Sciences, Liverpool School of Tropical Medicine, Liverpool, UK

The abstract of this study by Tenywa *et al* states that this study ‘intended to develop and test an ivermectin-based attractive-targeted sugar bait (ATSB) against *Aedes aegypti*, yet this does not reflect the results presented and it is important that the authors correct this. The results of the laboratory study demonstrate the killing effect of ivermectin fed in a sugar meal to male and female *Ae. aegypti*. The semi-field study suggests, despite issues with the experimental design which I discuss below, that *Ae. aegypti *released into a biodome in the absence (presumably, it is not mentioned) of competing sugar sources appeared to feed on sugar plus ivermectin in a prototype feeding station. The presence of devices with ivermectin over a 10-day period reduced the number (or percentage) of mosquitoes caught in daily collections relative to other time periods and the control compartment. Finally, the field experiment showed that free flying mosquitoes attempted to feed on stations inside garages and were caught on the sticky mesh lid of the device, and that a greater number of mosquitoes attempted to feed when a CO _2_ source was attached to the stations. Thus, the study demonstrates toxicity of ivermectin and the attractancy of CO _2_, using a prototype bait station which still has some serious development needs. Firstly, the stations seem to need to be replaced every 5 days, which is likely not feasible in full scale deployment of an intervention. There is also a safety issue and an environmental issue to non-target organisms to consider, with open containers of sugar water with a mesh grill kept at ground level with no means that I can see to protect children or other organisms from exposure.

 Whilst this is an early proof of principle that ivermectin could be used in an ATSB deployment strategy, there is very little evidence of efficacy of this prototype and certainly it does not represent a final product which could be deployed. The results of the laboratory and semi-field experiments are confounded in the Conclusion, since a 95% reduction in population was certainly not demonstrated in the semi-field environment, and the comment that the stations failed to reduce populations in the field experiment is false since the experimental design was not set up to show efficacy. It is essential that the work presented is framed correctly, to correctly reflect the results of the studies, and not overclaim the significance of the results – as it stands the conclusion is that an ATSB using ivermectin can only work with a CO _2_ source, which is a claim potentially damaging to innovation in the development of ivermectin-based ATSBs. Critically, this study shows that CO _2_ attracts mosquitoes to the stations, but not that it encourages feeding – stations may therefore mostly target blood feeding females who may not sugar feed, and without being confident that all of those females that are attracted feed and die you are potentially increasing the biting risk to people living nearby these stations. In reality, no attractant was included in the bait stations, and so it is inaccurate to label them ATSBs or expect them to compete with natural sugar sources and be effective in population control. On the subject of terminology, the Introduction refers to ‘Attractive Targeted Sugar Baits’, a name trademarked by Westham Co for their device. The generic term is ‘Attractive Toxic Sugar Baits’ which is more relevant here since there is nothing about the device that targets them to mosquitoes specifically.

 Beyond the framing and accurate representation of the results, which I strongly believe must be corrected, I think there are issues with the experimental design of the semi-field and field experiments, as well as the analysis and visualisation of data which should also be addressed.

 The major issue with the semi-field experiment is that the recapturing regime is problematic – 100 mosquitoes are released into the compartments at 6am, and at 6:30am a Bioagent trap is added to carry out collections and in phase 2 this included an additional HLC between 6am and 6:20am. There seems to be to be a high likelihood that a proportion of the captured mosquitoes would be the same ones which had just been released. It is thus confusing that 100% mortality was measured in the mosquitoes recaptured from the compartment with the toxic baits – unless the captured mosquitoes were screened for sugar feeding before mortality was scored, which should be mentioned in the Methods if so. In fact, a food dye was included in the experiments according to the Methods, but no data is presented on feeding rate for any experiment in the Results. Since there is no clear out of mosquitoes each day, with 50-60% of mosquitoes recaptured according to Figure 4 (if the Y axis does represent %, but in any case 100 mosquitoes were released every day), there would be 40-50% of mosquitoes remaining in the compartments from one day to the next, and so the populations would be expected to keep growing, unless there was 40-50% mortality in the compartments each day, which does not seem to match the data in Figure 4, or unless there was perfect recapture of all mosquitoes each morning which should be stated in the Methods if so.

 I also have concerns about the field experiment, which is essentially a demonstration of the bait stations as a sticky trap, with and without an attractant (CO _2_), and not a test of efficacy of the stations as seems to be claimed. There is no way to know which of the mosquitoes that were trapped would have fed on the sugar, nor any attempt to look at the effect on the population, particularly since the treatments were regularly swapped during the experiment. Given that, it’s a minor point, but with the large number and high density of stations in small sites, the risk of trapping out needs to be addressed – there isn’t a clear trend for reduced catches in the meta data but in many cases a large catch was followed by a lower catch the following day, particularly in garage 2. A major issue with this experiment is the inclusion of water controls and the way the data from them is handled. The captures in the water only controls were surely affected by the presence or absence of CO _2_ in the nearby stations, since CO _2_ will act as an attractant over an area of a few meters. Yet in the analysis the data from all water controls from both sites seems to have been combined, without accounting for the double trapping effort this constitutes. Doing some calculations from the meta data, when only compared against water on days when a competitor station was tested then ATSB alone is significantly more attractive than water, though still not as attractive as an ATSB with CO _2_. Thus it is not accurate to show, as in Table 2, that the bait without CO _2_ attracted as many mosquitoes as water alone.

 In several places the meta data does not match the results or methods presented in the paper – for example the text states that in the field experiment stations were swapped every 24 hours, but it seems from the meta data this was done every 3 days after day 1. The text states that the field experiment was conducted between November and December 2019, but the meta data suggests December 26 ^th^ to January 6 ^th^. This needs to be carefully checked and misrepresentations corrected.

 There has been a mistake made in the plotting of Figure 5, which does not match the text – the graph suggests only ~15% died in the ATSB compartment and 100% in the ASB compartment, whereas the text states 100% and 7.5% mortality, respectively. There is also an issue with the meta data presented for this experiment, where the sum values are incorrect in the summaries of the replicates. The numbers reported alive and dead in each replicate differs from the total numbers reported in the summary, so that the total number dead exceeds the number alive at the previous time point.

 More minor issues to be resolved:

 Methods:

- More information is needed about the mosquito colony used for the lab and semi-field experiments – origin, rearing details, quality control measures, insecticide resistance phenotype, any known resistance markers.
- In the first lab experiment and the semi-field experiment it is not clear whether male, female or both sexes were used.
- Why was a different range of concentrations used in the first and second lab experiments?
- It is not clear whether the stations tested in semi-field experiments included the sticky mesh lid, which is important to interpretation of the results and should be clarified.
- It would be interesting to compare the density of bait stations in the semi field experiment (5 stations in a 19x29m enclosure = 1 per 0.01 ha) and field experiment with the densities tested in published field trials of other ATSB products.
- Was there any vegetation or other natural sugar sources around the garages in the field experiment?
- In the field experiments, why were the treatment stations 10m apart, but the control stations at a distance of 3m?
- A lot more detail is needed in the data analysis section, and there are a few questions, for example, since compartment is a proxy for treatment in the semi-field experiment, why is it included as a fixed effect? Why is mosquito age included as a covariate when the same age of mosquito were used for all experiments?

Results:

- Figure 3 – without the inclusion of the 0% ivermectin data points it is not clear that there is a dose response in several of the groups. It is confusing the present the data by time point, and I would rather see time along the X axis and data plotted by concentration. As it is presented, the legend would be clearer if it was inverted to match the order of the lines on the graph.
- ‘ATSB efficacy against *Ae. aegypti *in a semi field environment’ – What do 35% and 56% recaptures represent, what’s the denominator?
- Figure 4 – what does the Y axis show, % or #, mean or total, recaptures just from traps or using HLC as well?
- Figure 5 – how can you be sure the exposure is 24 hours, when there are daily releases and daily recaptures, but no way of knowing how long those you recapture have been in the compartment? Unless there was perfect recapture of all mosquitoes each morning which should be stated in the methods if so. I suggest being consistent in terminology to avoid confusion – ASB is used in the figure and control-ATSB in the text. What does the dotted line at 0.5 represent?

Discussion:

- I am not sure that the data from lab experiments supports the first sentence regarding males and females being equally sensitive to ivermectin, since more males than females were killed within 24 hours at the lower concentrations.
- The dose response shown in Figure 3 is also not clear, and so as shown does not seem to support the second sentence.
- How do the volumes of blood ingested by a female compare to the volumes of sugar ingested by males or females? It would be interesting to factor this in when comparing results from endectocide studies and this study with sugar.
- ‘This study demonstrated that ATSB deployed inside a semi-field system remarkably reduced *Ae. aegypti* populations…’ – I am not sure the data support such a strong claim. Certainly there was a reduction in recaptured mosquitoes in Phase 2, but without knowing whether a stable population had been established in the biodome which was then reduced by the deployment of stations the exact effect is not clear.
- ‘The difference in survival time of the mosquitoes from the two groups suggests that the majority of the mosquitoes were attracted by the ATSBs and fed on it.’ – to justify this claim we would need to understand the method more clearly, for example were the recaptured mosquitoes scored for sugar feeding and mortality only measured in those that had fed? What numbers of fed v unfed mosquitoes were captured?
- ‘In the field, the ivermectin-based sugar bait stations were inefficient at attracting wild *Ae. aegypti*.’ – as discussed above, if the analysis was repeated with the water only controls separated by treatment there is some evidence of attractancy, albeit less than with the inclusion of CO _2_. Also as discussed above, there was no attractant included in the stations so I am not sure an attractive effect would have been expected. In this paragraph it would be useful to cite the recent paper about competition between ATSBs and natural sugar sources by Muyaga *et al., *(2023) ^1^.
- ‘mosquito may take a longer time to allocate the solution’ – this is not clear, do the authors mean to say ‘locate the station’?

Conclusion:

- Discussed above, this conclusion is not very clear as written, and I don’t believe it accurately represents the results from the study performed or their implications.

Is the work clearly and accurately presented and does it cite the current literature?

No

If applicable, is the statistical analysis and its interpretation appropriate?

Partly

Are all the source data underlying the results available to ensure full reproducibility?

Yes

Is the study design appropriate and is the work technically sound?

Partly

Are the conclusions drawn adequately supported by the results?

No

Are sufficient details of methods and analysis provided to allow replication by others?

No

Reviewer Expertise:

Rosemary Lees is a medical entomologist, with expertise in development and evaluation of vector control tools and mosquito control interventions, and development and validation of bioassay methodologies for the evaluation of insecticide-based products and for monitoring insecticide resistance. Daniel McDermott is a medical entomologist and statistician with an expertise in study design and evaluation of vector control interventions in semi field and community based trials.

We confirm that we have read this submission and believe that we have an appropriate level of expertise to state that we do not consider it to be of an acceptable scientific standard, for reasons outlined above.

## References

[1] : Effects of vegetation densities on the performance of attractive targeted sugar baits (ATSBs) for malaria vector control: a semi-field study. *Malar J* .2023;22(1) : 10.1186/s12936-023-04625-z 190 10.1186/s12936-023-04625-z 37344867PMC10283285

# Peer review: Reviewer response for version 1

**DOI:** 10.21956/wellcomeopenres.19287.r58211

**Published:** 2023-07-03

**Authors:** Fernando Ariel Genta

**Affiliations:** 1 Laboratory of Insect Physiology and Biochemistry, Oswaldo Cruz Institute - Oswaldo Cruz Foundation (IOC-FIOCRUZ), Rio de Janeiro, Brazil

The manuscript by Tenywa *et al.* brings a straightforward description of the application of ivermectin-based ATSB against *Aedes aegypti* mosquitoes. It is well-designed, and the results are very interesting, pointing out this strategy's potential and limitations. I have only very minor comments or requests.

 Introduction:

- "...as well as other sugar-questing vectors such as sand flies..." - Please include the new references on ATSB against sand flies provided, Ferreira *et al.* 2018 ^1^ and 2022 ^2^).
- "...a parameter that highly influences disease transmission" - Missing final dot.
- "...fitness and virus replication in the mosquitoes." - Remove "the".

Methods:

- "...they were fed cow blood through a membrane." - Please provide which anticoagulant was used and membrane.
- "... 100 *Ae. aegypti* mosquitoes aged 2 – 5 days, blood naïve and starved for 6–8 hours were released into each compartment" - Females/males only? Or mixed? Please clarify.

Results.

- "Sugar solution containing ivermectin 0.03% (w/v) caused approximately 80% of mosquito mortality for both mosquito sexes within 24 hours and > 90% within 48 hours (Figure 3)." - Data in Figure 3A does not fully support this sentence, the female 24h mortality is around 60% not 80%.
- Figure 3. Mean cumulative proportional mortality of female (A) and male (B) *Ae. aegypti* post feeding on 10% sugar solution that contained different ivermectin concentrations. - Please describe what is the meaning of the curves in the graph, in the figure legend. I see poor fitting in the 24 and 48h data. Is it possible to calculate a LD50 or LD90 using a probit-logit fitting tools?
- Figure 5. Effect of attractive targeted sugar bait on survival of female *Ae. aegypti *exposed to the baits in a semi-field system for 24 hours. - Please give the number of mosquitoes used for each curve. These are females or males?

Is the work clearly and accurately presented and does it cite the current literature?

Yes

If applicable, is the statistical analysis and its interpretation appropriate?

Partly

Are all the source data underlying the results available to ensure full reproducibility?

Partly

Is the study design appropriate and is the work technically sound?

Yes

Are the conclusions drawn adequately supported by the results?

Yes

Are sufficient details of methods and analysis provided to allow replication by others?

Partly

Reviewer Expertise:

Insect Biochemistry and Physiology

I confirm that I have read this submission and believe that I have an appropriate level of expertise to confirm that it is of an acceptable scientific standard.

## References

[1] : Effects of anti-Leishmania compounds in the behavior of the sand fly vector Lutzomyia longipalpis. *Pest Manag Sci* .2022;78(7) : 10.1002/ps.6900 2792-2805 10.1002/ps.6900 35411662

[2] : Transmission blocking sugar baits for the control of Leishmania development inside sand flies using environmentally friendly beta-glycosides and their aglycones. *Parasit Vectors* .2018;11(1) : 10.1186/s13071-018-3122-z 614 10.1186/s13071-018-3122-z 30501613PMC6271627

# Peer review: Reviewer response for version 1

**DOI:** 10.21956/wellcomeopenres.19287.r58210

**Published:** 2023-06-15

**Authors:** Tainá Neves Ferreira

**Affiliations:** 1 Laboratório de Biologia de Insetos (LABI), Universidade Federal Fluminense, Niterói, State of Rio de Janeiro, Brazil

**Abstract **

- *"Ae. aegypti were released into each cage and observed for mortality after 4, 8, 24, and 48 hours."* - Why do you check only until 48 hours? Please explain better why not evaluate more time.

**Methods**

Mosquitoes

- *"The mosquitoes were reared at 27±5ºC and 40%-99% humidity at Ifakara Health Institute insectary in Bagamoyo and Ifakara, Tanzania" - *Very high variation of temperature and humidity. Was this range just for lab tests or were they combined with data from semi-field tests as well? Try to describe it in more detail.
- *"Similar experimental setup and procedures as described above were performed for males (…)"* - Was the procedure described done with females? Please clarify in the text, as in other insects (kissing bugs, for example) the males also feed on blood.

Semi field experiment

- *"To maintain the quality of the control-ATSB and ATSB, both were replaced with a new one after every five days."* - Did you do any previous tests evaluating the stability of ivermectin mixed with sugar? How did you establish the exchange every 5 days? It is important to describe these data to ensure that the ivermectin is not being degraded or that there is loss through evaporation, since the temperature can reach 32ºC and the humidity is below 50%.

Field experiment

- *"Preparation of attractive targeted sugar bait (ATSB) with and without a carbon dioxide source.* *Three test bait stations were prepared as follows:* *1) control bait consisted of plain water dyed with 0.5% v/v red food coloring dye; 2) test-ATSB consisted of 10% w/v of sugar solution and ivermectin (final concentration 0.03% w/v);" - *Why was there no dye in the other baits? How to know if the mosquitoes were feeding on the test baits if the solution is colorless? Have you thought of using another dye in a different color?

Data analysis

- Explain a little more how these analyzes were carried out. It is not clear how the analyzes were done for users of other software.

**Discussion**

- *"The difference in survival time of the mosquitoes from the two groups suggests that most of the mosquitoes were attracted by the ATSBs and fed on them."* - With the use of another dye, you would be sure of the food.
- *"The vegetation spraying approach involves spraying the whole vegetation with a toxic sugar solution, whereas the bait station approach presents the toxic sugar bait at one single point. In this context, the bait station approach may hypothetically be regarded as less effective in attracting mosquitoes because mosquitoes may take a longer time to allocate the solution."* - This part could be enhanced with the works made by Günter C Müller and Yosef Schlein *et al.* There seems to be a missing reference in this paragraph.
- Did you manage to do any preference tests, even in the laboratory, to assess the preference for control bait (ASB) and toxic bait (ATSB) in the same cage or room? It would be important data to assess the preference of mosquitoes for the bait compared to other sources of natural sugars, especially in the absence of CO _2_. 

**Conclusions**

- *"We recommend further research to improve bait station design and attractant strategies such as plant-based volatiles."* - In addition, to plant volatile compounds, you could think of other more specific attractant compounds for mosquitoes or even for *Aedes aegypti* as pheromones. This would reduce limitations with non-target species.

Is the work clearly and accurately presented and does it cite the current literature?

Yes

If applicable, is the statistical analysis and its interpretation appropriate?

I cannot comment. A qualified statistician is required.

Are all the source data underlying the results available to ensure full reproducibility?

Yes

Is the study design appropriate and is the work technically sound?

Yes

Are the conclusions drawn adequately supported by the results?

Yes

Are sufficient details of methods and analysis provided to allow replication by others?

Partly

Reviewer Expertise:

Entomology, vector borne, diseases, parasite-vector interaction (invertebrate), vector control, parasitology  (trypanosomatids).

I confirm that I have read this submission and believe that I have an appropriate level of expertise to confirm that it is of an acceptable scientific standard, however I have significant reservations, as outlined above.

# Peer review: Reviewer response for version 1

**DOI:** 10.21956/wellcomeopenres.19287.r47841

**Published:** 2022-01-20

**Authors:** Gaurav Kumar

**Affiliations:** 1 ICMR-National Institute of Malaria Research, New Delhi, India

The manuscript evaluates ivermectin based sugar baits both under laboratory and field settings. But before consideration of manuscript for indexing, some clarifications are required from authors.

- In semi field conditions, approximately 35% and 56.8% mosquitoes were recaptured in compartment with ATSB and control-ATSB respectively. Which shows less attractiveness of ivermectin based sugar bait. Did authors test for the repellent effect of ivermectin, if any?
- After the introduction of Ivermectin-based sugar bait, there was merely a 38% decline in *Aedes* recapture rate. Authors should discuss it in comparison to other ATSBs with different toxicants like boric acid. The reduction rate does not seem as exciting as under natural field conditions, it may be lowered.
- Authors used term ATSB in which T stands for targeted while the common full form of ATSB is somewhat different. Authors should explain why they have done so.
- Moreover, no attractant was used in lab and field conditions therefore it should be toxic sugar baits (TSB) rather than ATSB.
- Authors should also explain the effect of Ivermectin sugar bait on male and female mosquitoes under filed settings. As Males may be targeted more by the baits.

Is the work clearly and accurately presented and does it cite the current literature?

Yes

If applicable, is the statistical analysis and its interpretation appropriate?

I cannot comment. A qualified statistician is required.

Are all the source data underlying the results available to ensure full reproducibility?

Yes

Is the study design appropriate and is the work technically sound?

Partly

Are the conclusions drawn adequately supported by the results?

Yes

Are sufficient details of methods and analysis provided to allow replication by others?

Yes

Reviewer Expertise:

Vector biology and control

I confirm that I have read this submission and believe that I have an appropriate level of expertise to confirm that it is of an acceptable scientific standard, however I have significant reservations, as outlined above.
